# Protocol of a randomized, single-blind, controlled trial of an 18-month, home-based, high-intensity, exercise intervention in older, cognitively unimpaired *APOE* ε4 carriers

**DOI:** 10.3389/fnagi.2025.1584115

**Published:** 2025-05-27

**Authors:** Stephen M. Rao, Alexandria L. Chorba, M. J. Helppi, Alina Tuladhar, Amanda L. Penko, Sarah Holley, Brandon Nehls, Sabrina Paulino, Matthew C. Streicher, Audrey Zhu, Kellie Bruening, Sally Durgerian, Katherine Koenig, Mark Lowe, Wanyong Shin, Stephen E. Jones, Rachel Galioto, Jagan A. Pillai, Lynn M. Bekris, James B. Leverenz, Anson B. Rosenfeldt, Tamanna Singh, Gerald J. Beck, J. Carson Smith, Jay Alberts

**Affiliations:** ^1^Center for Brain Health, Neurological Institute, Cleveland Clinic, Cleveland, OH, United States; ^2^Department of Biomedical Engineering, Lerner Research Institute, Cleveland Clinic, Cleveland, OH, United States; ^3^Concussion Center, Neurological Institute, Cleveland Clinic, Cleveland, OH, United States; ^4^Department of Quantitative Health Sciences, Lerner Research Institute, Cleveland Clinic, Cleveland, OH, United States; ^5^Department of Diagnostic Radiology, Imaging Institute, Cleveland Clinic, Cleveland, OH, United States; ^6^Mellen Center, Neurological Institute, Cleveland Clinic, Cleveland, OH, United States; ^7^Lerner Research Institute, Genomic Medicine Institute, Cleveland Clinic, Cleveland, OH, United States; ^8^Department of Cardiovascular Medicine, Heart, Vascular and Thoracic Institute, Cleveland Clinic, Cleveland, OH, United States; ^9^Department of Kinesiology, School of Public Health, University of Maryland, College Park, MD, United States

**Keywords:** Alzheimer’s disease, exercise, neuroprotection, APOE ε4, randomized clinical trial

## Abstract

**Background:**

The World Health Organization highlighted the potential protective role of exercise against cognitive decline, all-cause dementia, Alzheimer’s disease (AD), and vascular dementia in healthy individuals. We have previously shown that exercise is particularly beneficial for older, cognitively unimpaired apolipoprotein E4 (*APOE* ε4) carriers. A key unanswered question is whether a long-term, high-intensity aerobic exercise intervention initiated in a cohort of previously inactive older individuals at genetic risk for AD has neuroprotective properties.

**Design:**

CYCLE-AD is a randomized, single-blind, single-center, controlled trial of a home-based, high-intensity exercise intervention involving 150 older ε4 carriers (ages 65–80 years) who are healthy, cognitively unimpaired, and physically inactive. Participants are randomized into two groups: indoor cycling (IC) or usual and customary care (UCC) (target of 75 each). IC participants exercise 3×/week on an upright stationary cycle ergometer at a moderate-vigorous intensity for 18 months. Those in the UCC group are expected to maintain enrollment levels of activity.

**Outcomes:**

Comparison of IC and UCC groups on change in primary and secondary outcomes over baseline, 9-month, and 18-month evaluations. Primary outcomes are VO_2peak_ (Fitness), 5-trial total recall on the Rey Auditory Verbal List Learning Test (Episodic Memory), and total hippocampal volume derived from structural MRI (Brain Atrophy). Secondary outcomes include comprehensive neurocognitive and physical function test batteries, MRI scans including structural and functional connectivity measures, and blood-based biomarkers.

**Hypotheses:**

Over an 18-month interval, physically inactive ε4 carriers who engage in high-intensity aerobic exercise will demonstrate less cognitive decline and hippocampal atrophy than physically inactive ε4 carriers who did not participate in a formal exercise program.

**Conclusion:**

Successful demonstration of a scalable, home-based, high-intensity aerobic exercise intervention in altering the trajectory of AD pathophysiology and its effects on cognitive functioning will transform AD treatment, improve patient outcomes and quality of life, and reduce healthcare costs.

## Introduction

In 2019 the World Health Organization highlighted the potential protective role of exercise in preventing or slowing cognitive decline, all-cause dementia, Alzheimer’s disease (AD), and vascular dementia in healthy older individuals (World Health Organization [WHO], 2019). Our research (Smith et al., 2011; Woodard et al., 2012; Smith et al., 2014; Smith et al., 2016), and those of others (Head et al., 2012; Galle et al., 2023), suggest that exercise may be particularly beneficial for cognitively intact, healthy older individuals (ages 65–80 years) who are at genetic risk for AD based on having one or both apolipoprotein E4 (*APOE* ε4) alleles. Compared to ε4 elders self-reporting exercise ≥ 3 times per week, we demonstrated that ε4 carriers exercising < 3 times per week had significantly reduced fMRI activation of memory regions (Smith et al., 2011), altered radial diffusivity in white matter tracts (Smith et al., 2016), and significant declines in episodic memory (Woodard et al., 2012) and hippocampal volume (Smith et al., 2014) after an 18-month follow-up interval. Importantly, among ε4 non-carriers, no significant longitudinal changes in cognition and brain imaging were observed between physically active and inactive participants, suggesting that exercise has a specific neuroprotective role in delaying the progression of AD in ε4 carriers. Furthermore, these longitudinal group differences were observed over a relatively short 18-month follow-up interval, well within the time frame for examining the effects of an exercise intervention within the context of a randomized controlled trial (RCT).

A key unanswered question is whether a long-term, high-intensity aerobic exercise intervention initiated in a cohort of previously inactive older individuals at genetic risk for AD has neuroprotective properties. To address this question, we designed the CYCLE-AD (Cycling to Cease or Limit the Effects of Alzheimer’s Disease) RCT funded by the National Institute on Aging (R01 AG070736; S. Rao and J. Alberts, Co-Principal Investigators). The goal of the ongoing CYCLE-AD RCT is to recruit 150 participants, ages 65 to 80 years, who are healthy, cognitively unimpaired, and carry one or both copies of the *APOE* ε4 allele. All participants either do not exercise regularly or occasionally engage in low-intensity exercise (e.g., walking, yoga). Participants are randomly assigned to one of two groups (target of 75 each): (1) an indoor cycling (IC) group that participates in high-intensity aerobic exercise in their home using the stationary Peloton Indoor Cycle (New York, NY)^1^ or (2) a usual and customary care (UCC) group that is expected to maintain enrollment levels of activity. The IC group is expected to exercise 3 times per week (minimum 90 min/week) for 18 months. Participants in both groups are assigned accelerometers to track overall physical activity (step count) throughout the 18-month interval. The sample size and 18-month intervention duration were determined from our prior studies (Smith et al., 2011; Woodard et al., 2012; Smith et al., 2014; Smith et al., 2016).

CYCLE-AD has three primary outcome measures that are collected at baseline (pre-intervention) and 18 months (post-intervention). These outcomes include a measure of fitness (VO_2peak_), a cognitive measure of verbal episodic memory (Rey Auditory Verbal List Learning Test), and a measure of brain atrophy (total hippocampal volume) derived from structural MRI scans. The cognitive and MRI measures were derived from our prior longitudinal observational studies (Woodard et al., 2012; Smith et al., 2014). We hypothesize that, in contrast to the UCC group, the IC group will experience significant improvements in VO_2peak_ because of the exercise intervention. We predict that the UCC group will demonstrate significant reductions in verbal episodic memory and hippocampal volume over the 18-month interval due to the progression of AD pathophysiology; in contrast, the IC group will remain unchanged on these measures, reflecting the neuroprotective effects of high-intensity aerobic exercise.

The first CYCLE-AD participant was randomized in October 2021. The 150th and final participant will be randomized in early 2025, with the post-intervention follow-up testing completed by mid-2026. Shortly thereafter, an unblinded group comparison analysis of the primary and secondary outcomes will occur (see below). This paper details the CYCLE-AD methodology and discusses implications of potential results.

## Materials and methods

**FIGURE 1:**
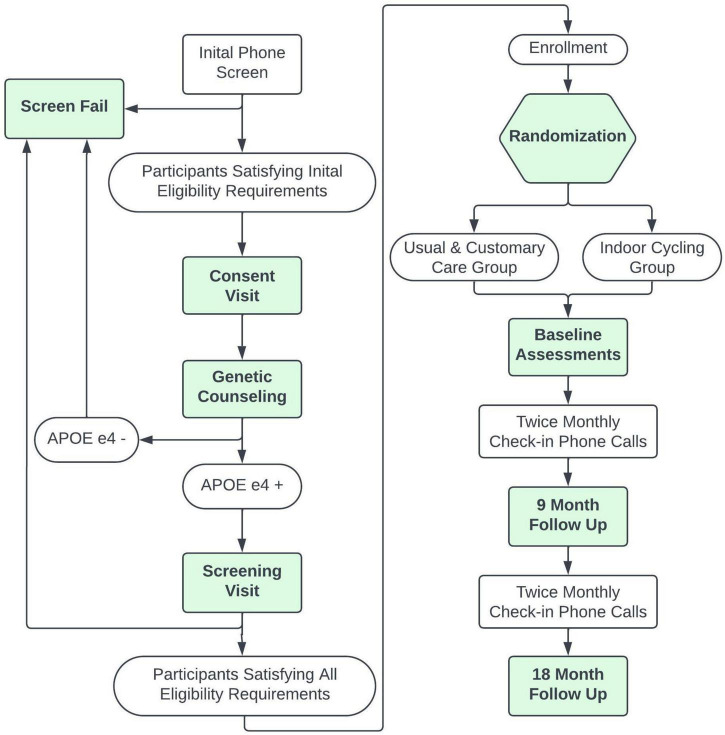
CYCLE-AD flow of study trial events.

Figure 1 provides a flow diagram summarizing the trial procedures detailed below and itemized in Table 1.

**TABLE 1:** Schedule of CYCLE-AD trial events.

Procedures	ICF visit	Screening visit (Within 90 days of ICF)	Baseline visit (Screening visit +30 days minimum)	9 month F/U (Day 0* + 9M ± 15 days)	18 month F/U visit (Day 0* + 18M + 30 days)
Informed consent	X				
APOE genotyping	X		X		
Telephone screening	X				
30-day pre-intervention physical activity		X			
Randomization and enrollment		X			
Cardiopulmonary exercise test (CPET)		X			X
Computer Assisted Rehabilitation Environment System			X		X
3T magnetic resonance scanning			X		X
Plasma biomarker collection/analyses			X		X
Indoor cycle fitting		X			
Peloton-garmin monitoring check-in			X	X	X
**Cognitive screening tests**					
Mini-mental status examination (MMSE)		X			
Wechsler Memory Scale-IV Logical Memory Delayed Recall		X			
Geriatric Depression Scale		X			
Self-Maintaining and instrumental ADL Scale		X			
**Clinical laboratory tests**					
Composite blood count with differential			X	X	X
B12			X		
Comprehensive metabolic panel			X		
Lipid panel			X		
Westergren sedimentation rate			X	X	X
C-reactive proteins			X	X	X
**Physical function test battery**					
Timed up and go			X		X
10 meter walk test			X		X
5× Sit to stand			X		X
6 minute walk test			X	X	X
**Questionnaires**					
World Health Organization Quality of Life–Old Module			X		X
Center for Epidemiologic Studies Depression Scale			X	X	X
Geriatric Anxiety Scale			X	X	X
Short form health survey (SF-36)			X		X
Emotion Regulation Questionnaire			X		X
Toronto Alexithymia Scale			X		X
Self-Efficacy for Exercise Scale			X		X
**Neurocognitive test battery**					
Wide Range Achievement Test 4, Reading			X		
Rey Auditory Verbal Learning Test (RAVLT)			X	X	X
Brief Visual Memory Test–Revised (BVMT-R)			X	X	X
Stroop Color and Word Test			X	X	X
Controlled Oral Word Association Test			X	X	X
Trail Making Test (A and B)			X	X	X
Delis Kaplan Executive Function System (DKEFS)–Sorting			X		X
Rey–Osterrieth Complex Figure Copy Test			X		X
Boston Naming Test			X		X
Judgement of Line Orientation			X		X
Processing Speed Test			X	X	X
Visual Memory Test			X	X	X
Wechsler Adult Intelligence Scale (WAIS) IV, Digit Span			X	X	X

*DAY 0 = ≤ 30 days of completion of baseline visit.

### Participants

#### Inclusion criteria

The four primary inclusion criteria are: (1) APOE ε4 carrier (ε2/ε4, ε3/ε4, or ε4/ε4) determined initially by a saliva swab kit and confirmed with a blood test, (2) ages 65 to 80 inclusive, (3) no evidence of cognitive impairment based on neurocognitive screening tests (see below), and (4) an inactive physical activity level, defined as exercising less than 3 times per week at moderate-vigorous intensity. In addition, the potential participant must be fluent in English, a requirement for neuropsychological testing; have no plans to travel for more than 2 consecutive weeks during the 18-month intervention; and have in-home WIFI to connect to Peloton content and transmit exercise data.

#### Exclusion criteria

Potential participants will be excluded if they exhibit: (1) any significant neurologic disease (including dementia and MCI), (2) significant medical illnesses/conditions (lung or kidney disease, cancer), (3) a history of schizophrenia or bipolar disorder, (4) Major Depression within the past year, (5) a history of alcohol or substance abuse or dependence within the past 2 years, (6) a current use of Alzheimer’s disease medications, including donanemab, lecanemab, cholinesterase inhibitors, and memantine, (7) any unstable or severe cardiovascular disease or asthmatic condition, (8) a history of imaging confirmed transient ischemic attack or a score of > 4 on the modified Hachinski ischemic scale (Hachinski et al., 1975), (9) significant abnormalities in clinical laboratory blood tests considered to be a contraindication for exercise, (10) exclusion criteria specific to MR scanning (weight inappropriate for height, ferrous objects within the body, pregnancy, and a history of claustrophobia), (11) a musculoskeletal disorder (e.g., arthritis, osteoporosis) that would limit the person’s ability to engage in high-intensity exercise, (12) inability to safely engage in high-intensity exercise, based on the American College of Sports Medicine [ACSM] (2014) exercise pre-participation screening, and (13) an abnormal cardiopulmonary exercise test (CPET; see details below).

#### Recruitment

We are employing two primary recruitment methods. The first involves a query of the electronic medical records of primary care patients followed by the Cleveland Clinic Internal Medicine and Family Medicine departments. Invitation letters are sent to primary care patients who have reported a first-degree family history of AD/dementia. By focusing on primary care patients with a family history of dementia, we aim to increase the percentage of carriers from 20% (general population) to 35%–40%, thus reducing the number of screen failures based on genotype.

The second method involves the recruitment of participants enrolled in the GeneMatch Alzheimer’s Prevention Registry (Langbaum et al., 2019), a national program overseen by Banner Health. All participants in the registry have undergone *APOE* genotyping, although they may not be aware of their genetic status. The GeneMatch program sends electronic and postal mailings to registry participants describing the CYCLE-AD project. GeneMatch provides Cleveland Clinic study staff contact information of individuals who have expressed interest in volunteering for the CYCLE-AD trial. Approximately 90% of the GeneMatch participants referred as potential participants to CYCLE-AD are ε4 carriers (10% non-carriers are included in the sample to minimize disclosure of genetic status for those GeneMatch participants unaware of their *APOE* test results). We are conducting nationwide recruitment from the GeneMatch registry; travel reimbursements are provided for participants who reside outside the metropolitan Cleveland area.

The decision to recruit 150 ε4 carriers is based on a sample size and power analysis described in the “Statistical analyses” section below. Additionally, our goal is to recruit 15 participants (10% of total recruitment) from minority populations.

### Screening procedures

#### Telephone screening

All volunteers undergo a standardized telephone screening to review medical and psychiatric history to determine if they meet inclusion and exclusion criteria. As noted above, the ACSM exercise pre-participation screening (2014) is administered to determine if the volunteer is safe to engage in high-intensity exercise.

#### APOE genotyping

Consented volunteers meeting inclusion/exclusion criteria during the telephone screening complete an oral swab test kit for preliminary APOE genetic testing. Genomic DNA is extracted from a 500 μl aliquot of saliva sample collected using an Oragene kit (DNA Genotek Oragene-DISCOVER: OGR-575). 20 μl of DNA Genotek prepIT-L2P purifier reagent (PT-L2P) is added to the sample, which is then pelleted and washed using ethanol. The sample is rehydrated with 100 μl of TE Buffer and stored at −20°C for long-term storage.

Confirmatory genotyping from blood testing is conducted only for those in which the saliva swab test results indicate they possess one or both ε4 alleles. Genomic DNA is extracted from participant blood samples collected during the in-person screening assessment (see below) using the Promega ReliaPrep LV HT gDNA Isolation kit (catalog # A2751) and the KingFisher Flex Purification System (Thermo Fisher Scientific) per manufacturer’s instructions. The resulting gDNA was assessed for quantity and quality using the Nanodrop One spectrophotometer and the Qubit 3 fluorometer (Thermo Fisher Scientific), respectively.

For both the oral swab and confirmatory blood tests, APOE genotyping was performed using the 7500 Real Time PCR System and TaqMan SNP Genotyping Assays (rs429358, rs7412) provided by Thermo Fisher. Each predesigned TaqMan assay includes two allele-specific TaqMan MGB probes that contain distinct fluorescent dyes and a PCR primer pair to detect the SNP targets. Each assay is run with three NTC wells alongside the unknown samples. The genotypes were determined (based on sample clustering) using the auto-caller function of the Genotyping Application within the browser-based Thermo Fisher Connect software.

All volunteers undergoing APOE genotyping are informed of the results through a genetic counseling session conducted by senior project clinicians.

#### In-person screening assessment

Volunteers who possess one or both ε4 alleles are invited to undergo an in-person screening assessment at the Cleveland Clinic. This assessment includes (1) a cardiopulmonary exercise test (CPET), (2) an evaluation to determine if the participant is capable of mounting and dismounting an indoor cycle, and (3) administration of an abbreviated psychometric examination that includes the Mini-mental status exam (MMSE) (Folstein et al., 1975), Wechsler Memory Scale-IV (WMS-IV) Logical Memory (Wechsler, 2009) delayed recall, Geriatric Depression Scale (GDS)–short form (Sheikh and Yesavage, 1986; Yesavage, 1988), and Self-Maintaining and Instrumental Activities of Daily Living Scale (ADL) (Lawton and Brody, 1969). The psychometric evaluation is conducted to ascertain whether the participant is cognitively unimpaired, not experiencing significant depression, and capable of performing ADLs necessary for exercise. Participants are excluded if they score < 27 on MMSE, < 6 scaled score on WMS-IV Logical Memory Delayed Recall (DR stories combined), > 19 on GDS, and < 4 on ADL scale.

### Intervention procedures

#### Randomization

Participants meeting inclusion/exclusion criteria and cleared for exercise by a cardiologist based on CPET results are randomized to either the IC or UCC group (*n* = 75 each), using a permuted block randomization, with random block sizes of 4 and 6, preloaded into a secure REDCap electronic data management system. All study personnel involved in outcomes assessments are blind to the participant’s group assignment.

#### Indoor cycling intervention group

Individuals in the IC group are provided a Peloton Indoor Cycle, which is delivered to their homes. They are also provided an 18-month subscription to the Peloton Platform. The commercially available Peloton Indoor Cycle was selected because it has an interactive platform that facilitates engagement by providing thousands of live and on-demand cycling classes. Classes vary in length, difficulty, and format (i.e., instructor-led, self-led, scenic, gamified), offering options to all fitness levels and interests. Additionally, the platform allows the monitoring and storing of exercise performance data [e.g., frequency, duration, cadence, and heart rate (HR)] for all exercise sessions. Participants wear a chest-worn or wrist-worn HR monitor that continuously transmits via Bluetooth to the exercise cycle. Performance metrics are continuously displayed on the monitor for instantaneous performance feedback. Summary exercise performance metrics for each ride are downloaded weekly via a custom script by a member of the study team.

Resting HR and maximal observed HR derived from the baseline cardiopulmonary exercise test (see below) are used to provide a participant-specific target HR range training zone, defined as 60%–90% of heart rate recovery (HRR) calculated using the Karvonen formula (American College of Sports Medicine [ACSM], 2018). The American College of Sports Medicine considers this zone high-intensity (American College of Sports Medicine [ACSM], 2018).

The aerobic exercise intervention is monitored by an exercise physiologist, who uses the exercise performance data from exercise sessions over a two-week period to aid in exercise progression and discuss potential exercise facilitators/barriers. Based on the inclusion criteria, all participants are physically inactive prior to enrollment; thus, participants are prescribed initial rides of 10 or 15 min at low to moderate intensity. If objective performance data from the Peloton platform demonstrate the participant is completing the 10–15 min rides, exercise is progressed in 5-to-10-min increments every 2–4 weeks, or as subjective and objective data indicated, until the prescribed 30 min per session, 3×/week at 60%–90% of their HRR, is achieved. This target exceeds the minimum guideline of 75 min per week of vigorous-intensity aerobic physical activity recommended for older adults by the US Department of Health and Human Services (USDHHS, 2018). Participants are encouraged to have a day of rest between exercise sessions. After the goal of 30 min of cycling exercise, 3×/week is achieved, participants are not limited to these exercise constraints and, if desired, can exercise with longer durations (e.g., 45 min) and greater frequency (e.g., 4–5×/week) if tolerated.

#### Usual and customary care group

Participants randomized to the UCC group are asked to continue their habitual level of physical activity established prior to their study participation. Participants in the UCC group are asked not to initiate a high-intensity exercise program (e.g., cycling, running, rowing) during the 18-month study duration. They are allowed to engage in low-intensity exercises (e.g., walking, yoga, resistance training).

#### Remote monitoring

Study team members call participants in both the IC and UCC groups two times per month to inquire about activity levels, changes in medication and health status, including possible adverse events, and technology assistance with the Garmin and/or Peloton device and data. For those in the IC group, exercise recommendations are discussed based on a review of prior cycling performance.

### Baseline and follow up assessments

Enrolled participants complete three in-person outcome assessment visits: (1) Pre-Intervention Baseline (0-month), (2) Mid-Intervention (9 months), and (3) End-of-Intervention (18 months). The baseline and 18-month visits are typically conducted over two days and are scheduled within two weeks of each other. The abbreviated 9-month visit is conducted over a single day. Tests performed during each visit are shown in Table 1 and described below. The total study participation time is approximately 19 months, extending from a 30-day physical activity pre-intervention baseline (see below) to the post-intervention 18-month follow-up assessment. Primary and secondary outcomes for the CYCLE-AD trial are listed in Table 2.

**TABLE 2:** Primary and secondary outcomes for CYCLE-AD trial.

Outcomes	Exercise	Cognition	MRI
Primary	Cardiopulmonary exercise test (VO_2peak_)	RAVLT (total recall trials 1–5)	Anatomical MRI (total hippocampal volume)
Secondary	CAREN biomechanical gait (velocity in m/s)	Episodic memory: RAVLT delayed recall; BVMT-R, Visual Memory Test	Anatomical MRI (hippocampal subfields)
	CAREN postural stability (sway area in cm^2^)	Processing speed: Trail Making Test A, WAIS IV Digit Span, Processing Speed Test	Resting-state functional connectivity (between hippocampus and posterior cingulate)
	Physical function battery (time up and go, 10 meter walk, 6 minute walk test, and 5× sit to stand)	Executive functions: DKEFS (sorting), Stroop Color and Word Test, Trail Making Test B	Diffusion tensor imaging (anatomical connectivity between hippocampus and posterior cingulate)
	Garmin Vivofit 4 Health Monitor (number of steps)	Visuospatial abilities: Judgement of Line Orientation, Rey–Osterrieth Complex Figure Copy Test	Three-dimensional arterial spin labeling (hippocampal and cortical gray matter blood flow)
	Self-report quality of life questionnaires	Language: Boston Naming Test, Controlled Oral Word Association Test	Fluid Attenuated Inversion Recovery (white matter hyperintensities)

#### Physical activity (PA) monitoring

All enrolled participants are provided a Garmin Vivofit 4^2^ device to measure steps per day throughout the trial. Participants are instructed to wear the device on their ankle (with a provided band) during waking hours for the entire 18-month trial duration. Additionally, PA baseline monitoring is obtained by having participants wear the device for 30 days before the baseline assessment. Participants are asked to synchronize the device with the Garmin Connect application installed on their mobile device or tablet on a weekly basis. Project personnel pull data from the Garmin portal weekly with an automated script to a secure HIPAA-compliant server behind the Cleveland Clinic firewall. The transfer process is completed weekly for each participant and stored on a secure shared drive. PA monitoring for both groups is supervised by an exercise physiologist.

#### Exercise fitness assessment

A comprehensive exercise/fitness assessment is conducted twice, at baseline pre-intervention and 18-month post-intervention assessment sessions. An abbreviated assessment occurs at 9 months (see Table 1). The comprehensive assessment has four components:

##### Cardiopulmonary exercise test (CPET)

The CPET is administered as part of the screening/baseline assessment and is repeated at the 18-month follow-up. It provides a measure of maximal oxygen consumption, VO_2peak_, an index of cardiorespiratory function and aerobic fitness. Participants are asked to refrain from food and drink for 4 h, except clear liquids, and abstain from caffeine for 12 h before the test. A resting 12-lead ECG is obtained initially in the supine and standing positions to determine possible contraindications to exercise testing. Participants are fit with a mouthpiece and nose clips for the measurement of cardiopulmonary data. All participants complete a maximal graded exercise test with gas analysis to measure peak aerobic capacity. The CPET is completed on an upright stationary cycle ergometer utilizing an individualized ramp protocol with the ramp set to elicit a test duration of approximately 8–12 min. Cardiopulmonary data is assessed via calibrated open-circuit spirometry. During the CPET, participants are instructed to maintain a consistent cadence throughout the entire test as the workload increases. The cycle ergometer ramp test increases watts by 12.5, in a ramp fashion, every minute until the participant reaches volitional exhaustion or satisfies the stopping criteria from the American Heart Association/American College of Sports Medicine. A 12-lead electrocardiogram is continuously monitored, and manual blood pressures are taken during the last 30 s of each stage of the test. Two criteria establish maximal effort: (1) a respiratory exchange ratio (CO_2_/O_2_) greater than 1.10, and (2) a Borg rating of perceived exertion greater than 7 on a 10-point scale. The CPET is a primary CYCLE-AD study outcome (Table 2).

##### Biomechanical gait analysis

The Computer Assisted Rehabilitation Environment (CAREN) system (Motekforce Link, Amsterdam, Netherlands) is used to quantify gait and postural stability (Baron et al., 2018; Penko et al., 2018; Rosenfeldt et al., 2019a; Rosenfeldt et al., 2019b). The system consists of a fully integrated Vicon 3D motion capture system, a treadmill, force plate platform, a 180° curved projection screen, and a safety harness. The modified Plug-In Gait marker model is used with 35 retro-reflective markers placed on anatomic landmarks. The 10-camera Vicon system, in combination with D-Flow software, captures the 3D position of each marker at 100 Hz. The primary gait measure is self-paced gait velocity (m/s) over a 1-min walk test with and without performing a secondary cognitive task, enabling an analysis of dual-task performance. Secondary measures include step length, step width, cadence, and arm swing (Baron et al., 2018). The limits of stability (LOS) test is a secondary outcome used to evaluate postural control (Pickerill and Harter, 2011) under single- and dual-task conditions. The force plate in the CAREN system is used to quantify the maximum distance an individual can displace their center of mass within their base of support in eight different directions. The primary postural control outcome is the sway area, the center of pressure area (cm^2^) throughout an 80-s trial.

##### Physical function testing

Participants complete a 6-minute walk test (6MWT). The primary measure is the total distance walked in 6 min. Heart rate and ratings of perceived exertion are also measured. Functional mobility is measured using the Timed Up and Go (Miller Koop et al., 2018; Miller Koop et al., 2019), 10 meter walk, and 5× sit to stand tests, which are completed using validated iPad applications (Ozinga and Alberts, 2014; Ozinga et al., 2015; Ozinga et al., 2017a; Ozinga et al., 2017b). Briefly, the iPad is attached at the sacral level of the participants during the performance of these clinical tests. Data gathered from the iPad IMUs (accelerometer and gyroscope) are used to calculate biomechanical measures of gait, postural stability, and specific phases (including turning velocity) of mobility assessments.

##### Quality of life questionnaires

Quality of life is assessed with the World Health Organization Quality of Life for Older Adults (WHOQOL-OLD) (Power et al., 2005). The Self-Efficacy for Exercise Scale is used to assess the ability to continue exercising in the face of barriers (Resnick and Jenkins, 2000).

#### Neurocognitive assessment

The standardized neuropsychological test battery consists of measures of premorbid intelligence, episodic memory, information processing speed, executive functions, and sustained attention. The test battery includes the Wide Range Achievement Test 4 (WRAT4) (Wilkinson and Robertson, 2006), Rey Auditory Verbal Learning Test (RAVLT) (Rey, 1958), Brief Visuospatial Memory Test-Revised (BVMT-R) (Benedict, 1997), Delis Kaplan Executive Function System (sorting test) (Delis et al., 2001), Stroop Test (Golden and Freshwater, 2002), Controlled Oral Word Association Test (COWAT) (Benton et al., 1983), Boston Naming Test (BNT) (Kaplan et al., 2001), Trail Making Test A and B (Reitan, 1958), Judgement of Line Orientation (JOLO) (Lindgren and Benton, 1980), Wechsler Adult Intelligence Scale IV (WAIS-IV) Digit Span (Forward, Backward, and Sequencing) (Wechsler, 2008), Rey-Osterrieth Complex Figure Copying Test (Rey, 1941; Osterrieth, 1944), Processing Speed Test (Rao et al., 2017), and Visual Memory Test (Rao et al., 2023). Where available, counterbalanced, alternate equivalent forms are used to minimize practice effects. The 5-trial total recall on the RAVLT is a primary outcome for the CYCLE-AD trial (Table 2).

#### Magnetic resonance imaging

MRI is conducted on a Siemens Prisma 3T MRI scanner with a 32-receive channel head coil. Participant motion is minimized using a bite bar. A molded dental impression (Kerr Dental, Inc., Brea, CA, USA) is taken of the subject’s teeth. This mold is affixed to a plastic frame placed over the head coil. The subject is asked to place their teeth into the mold during active scanning. Including patient preparation and scanner tuning, this protocol is accomplished in 90 min. Preliminary scans include a localizer and two -GRE B0 field maps. Detailed scan parameters are provided in Table 3. The following section provides information on the collection and analysis of each scan.

**TABLE 3:** Detailed scan parameters for MRI protocol.

Scan						
**Sequence parameter**	**3D T1**	**High Resolution Hippocampus**	**3D FLAIR**	**CBF**	**Resting State fMRI**	Diffusion MRI*
Sequence	MPRAGE	Turbo Spin Echo	SPACE	3D Pulsed ASL	SMS GRE-EPI	SMS-SE-EPI
Scan plane	Sagittal	Coronal	Axial	Axial	Axial	Axial
Time of acquisition (min:sec)	5:21	4:18	5:17	5:40	6:01	7:55
FOV (mm^2^)	256′256	175′175	256′256	240′240	260′260	220′220
Matrix	256′256	448′448	256′256	64′64	104′104	110′110
Voxel size (mm)	1′1′1	0.4′0.4′2	1′1′1.2	3.75′3.75′4.5	2.5′2.5′2.5	2′2′2
TI	900 ms	N/A	1,800 ms	800/2,000 ms	N/A	N/A
TE	2.98 ms	52 ms	393 ms	20.25 ms	29 ms	75 ms
TR	2,300 ms	8,000 ms	5,000 ms	4,000 ms	1,360 ms	2,700 ms
Flip angle	9°	150°	variable	180°	65°	90°
Receiver bandwidth (total)	61.44 kHz	17.675 kHz	200 kHz	156 kHz	227 kHz	250 kHz
Partial Fourier	N/A	N/A	N/A	N/A	8-Jul	8-Jun
GRAPPA	2	2	2	none	None	none
(ACC/ACS)						
SMS	N/A	N/A	N/A	N/A	3	3

*Additional information for dMRI: 8/30/17/60 diffusion weightings of 0/7/1,000/2,800 s/mm^2^ b = 2,800 s/mm^2^ are acquired. Following the diffusion acquisition, an 18 s phase-reversed b = 0 volume is acquired to be used for post-processing distortion correction.

##### Anatomical imaging (3D T1-weighted scan)

Total hippocampal volume (right plus left) is a primary outcome for the CYCLE-AD trial (Table 2) and is calculated using the MPRAGE in Freesurfer (7.4.1) (Iglesias et al., 2015). Individual differences in head size are corrected for by allometric scaling (Liu et al., 2014) or by using log(ICV) as an adjustment regressor (Williams et al., 2021). In addition, the ADNI high-resolution hippocampal scan is used to calculate hippocampal subfield volumes in ASHS (ashs-1.0.01) (Xie et al., 2016). Measures of interest include the sum of CA1, CA2, and CA3, the dentate gyrus, and the subiculum.

##### Resting-state functional connectivity (rs-fMRI)

Functional connectivity between the hippocampus and the posterior cingulate is measured using the resting-state connectivity scan. During scanning, respiratory and cardiac signals are sampled at 400 Hz using a plethysmograph and respiratory bellows. These signals are regressed using RETROICOR (Glover et al., 2000) as provided by AFNI. If physiologic signals are not recorded, they are estimated using PESTICA (pestica_afni_v5.521) (Beall and Lowe, 2007). Volumetric and slice-based motion are estimated and corrected using SLOMOCO (slomoco_afni_v5.52) (Beall and Lowe, 2014; Shin et al., 2022). Motion and physiologic noise estimates are regressed simultaneously. An estimate of voxel-level residual displacement is used to characterize motion artifact in each scan for use in summary statistics and quality control. The measure of interest is the normalized strength of functional connectivity measured between the posterior cingulate seed and the hippocampus (Koenig et al., 2021a).

##### Diffusion tensor imaging (DTI) anatomical connectivity

Structural connectivity of the hippocampus and posterior cingulate is measured using a DTI scan. Preprocessing includes distortion correction with topup and motion (Andersson and Sotiropoulos, 2016), and eddy current (Jenkinson et al., 2012) correction (FSL 6.0.7.12). The left and right ventral cingulum bundles are identified using Freesurfer 7.4.1 and tracked using TRACULA (Maffei et al., 2021). Measures of interest include average axial and radial diffusivity along the bilateral ventral cingulum bundle.

##### Three-dimensional arterial spin labeling (3DASL)

Hippocampal and cortical gray matter blood flow is calculated using the 3DASL scan. Data are preprocessed using AFNI (AFNI_24.0.02) (Cox, 1996) and oxford_asl (FSL 6.0.7.12) (Chappell et al., 2009). The hippocampal region of interest (ROI) is taken from the Freesurfer analysis. The posterior cingulate ROI is extracted from the Yeo 7 Network parcellation of the default mode network (Yeo et al., 2011). Voxels are restricted to those composed of ≥ 70% gray matter. Measures of interest are the average perfusion in ml blood / 100 g tissue / min. in each ROI.

##### White matter hyperintensities (WMHs)

WMH volumes are calculated from T2 Fluid Attenuated Inversion Recovery (FLAIR) scans. WMHs are calculated using the LST-AI software^3^ (Wiltgen et al., 2024). The measure of interest is lesion volume in mm^3^.

#### Blood biomarker analyses

All blood samples are collected in the morning after fasting for 12 h. Plasma analyses (Weber et al., 2020; Koenig et al., 2021b; Weber et al., 2022) include measurements of anti-inflammatory (IL-4, IL-6, IL-10, IL-13, sTREM2), pro-inflammatory (IFN-γ, TNF-α, IL-1β, IL-6, MCP-1), neuronal (BDNF, NFL, NSE, Neurogranin), and AD-related (Abeta40, Abeta42, p-tau181, t-tau) biomarkers. Changes in biomarkers over the 18-month intervention interval serve as tertiary outcome measures.

### Statistical analysis

#### Sample size and power

The CYCLE-AD RCT will compare changes from baseline to 18 months on the primary and secondary outcomes (see Table 2) in the UCC and IC randomized groups (75 per group). The sample size was estimated for each of the three primary outcomes: (1) trial 1–5 total recall on the RAVLT (RAVLT_*T1–5*_), (2) total hippocampal volume (THV), and (3) VO_2peak_. To determine the sample size necessary to detect a clinically meaningful difference between the groups for each primary outcome, we first estimated the variability (standard deviation) of the changes over 18 months in each variable from previous studies. Data for RAVLT_*T1–5*_ and THV are derived from our 18-month longitudinal study comparing high and low PA APOE ε4 carriers (Woodard et al., 2012; Smith et al., 2014); data for VO_2peak_ are derived from a published study (King et al., 1991). For each outcome, we calculated the pooled SD (combining intervention groups), and since the pooled SDs are only an estimate of the variability of changes that were observed from each study, we increased the pooled estimate to be equal to the upper 90th percentile of the SD distribution for each primary outcome. The assumed pooled and inflated SDs are: 17.15% for RAVLT_*T1–5*_, 4.35% for THV, and 3.16 ml/kg-min for VO_2peak_. Secondly, the size of the clinically meaningful difference to be detected between the groups for each outcome was determined based on the observed values seen in these previous studies. The differences to detect for changes over 18 months are: 9.5% for RAVLT_*T1–5*_, 2.4% for THV, and 1.75 ml/kg-min for VO_2peak_. These differences are similar to those actually observed in the cited studies and were selected to give similar sample sizes so that each aim would have adequate power with the same sample size. Hence, the detectable differences and SDs given above resulted in an estimated sample size for each primary outcome of 68 per group, assuming a power of 90% and a significance level of 5% (two-sided). Furthermore, we estimated that up to 10% of participants will not complete the 18-month measurements. Therefore, we inflated the sample size of 68 per group by 10%, yielding a study sample size target of 75 per group.

#### Data analysis

The primary outcome analyses follow the Intent-To-Treat principle, with all randomized participants included in the final analyses. If persons are missing the 18-month measurement so that no change over 18 months can be computed, we will impute their 18-month value. An As-Treated analysis is done as a secondary analysis. The UCC and IC groups will be initially characterized and compared on baseline demographic, medical comorbidities, and baseline PA/fitness variables, using frequencies and proportions for categorical variables, means and standard deviations for continuous symmetric confidence intervals, and Tukey five-number summaries for continuous skewed variables. If any clinically significant differences between groups appear despite randomization, an adjustment will be considered in the sensitivity analyses.

The primary analysis compares the UCC and IC groups on mean changes from baseline to 18 months on the three primary outcomes: RAVLT_*T1–5*_ (memory), THV (hippocampal volume), and VO_2peak_ (fitness). This analysis will use a *t*-test unless the distribution of changes is non-normally distributed, in which case the non-parametric Wilcoxon rank sum test will be used. Baseline covariates may be included in the primary analysis using an ANCOVA to reduce noise and consequently increase power to protect against concerns of non-negligible confounding due to post-randomization treatment group imbalances or both. These covariates will be pre-specified and include factors used for subgroup analyses, such as sex, race, age, and education. Similar analyses will be performed on secondary outcomes (Table 2) but will be labeled as data-driven, exploratory, and hypothesis-generating.

Subgroup analyses will give descriptive summaries of the primary and secondary outcomes (see Table 2) stratified by sex, race, education, and age (ages 65–72 and 73–80). Other subgroups may be defined based on baseline health variables (hip/waist ratio circumference, resting heart rate, blood pressure) and 30-day pre-intervention Garmin Vivofit 4 physical activity monitoring levels. These pre-specified subgroup analyses will be used to detect evidence for modification of the exercise treatment effect by each of the above covariates. These analyses will be done by testing the statistical significance of the interaction of each potential effect modifier with treatment. Conventional forest plots will be used, regardless of the outcome of statistical testing, to display point estimates and 95% confidence intervals for exercise effects of each potential effect modifier. These analyses will be reported as data-driven, exploratory, and hypothesis-generating.

Regression models will be developed in several domains to explore what factors may be associated with 18-month changes in the three primary outcomes: RAVLT_T1–5_, THV, and VO_2peak_. An example would include changes in blood-based anti-inflammatory, pro-inflammatory, neuronal, and AD-related biomarkers. For the IC group, primary outcome changes will be regressed with stationary bike performance over time: compliance, exercise duration, time in the target heart rate zone, and cadence. Given the limitations imposed by our sample size, these regression analyses will be considered exploratory but have the potential to provide valuable information regarding factors to consider in developing prediction models for future larger follow-up studies designed to more fully examine the neuroprotective mechanisms associated with high-intensity exercise in older elders at genetic risk for AD.

## Discussion

The CYCLE AD RCT was designed to parallel our 18-month longitudinal observational studies (Smith et al., 2011; Woodard et al., 2012; Smith et al., 2014; Smith et al., 2016) comparing low-exercise (< 3 times per week) versus high-exercise (≥ 3 times per week) ε4 carriers. The neuroprotective properties of exercise that we observed in brain atrophy and cognition may have resulted from lifelong habitual levels of exercise. A recent epidemiological study, however, noted that low-exercise individuals who become fit by increasing their exercise levels reduced their chances of developing dementia, similar to those who were fit for most of their lives (Tari et al., 2019). The CYCLE-AD trial, therefore, will test the concept that a high-intensity aerobic exercise intervention initiated later in life can maintain CNS function and offer neuroprotection.

A unique feature of the CYCLE-AD trial is the long duration of the exercise intervention (18 months). In a 2019 meta-analysis (Falck et al., 2019), Falck et al. reviewed 56 exercise intervention studies and noted that the median intervention duration was a relatively brief 4.6 months (75th percentile < 9.5 months). Meta-analyses suggest that longer exercise program durations in clinical trials are associated with greater gains in fitness (VO2max) (Silva et al., 2014; Borde et al., 2015; Oja et al., 2018) and maintenance of cognition (Colcombe and Kramer, 2003) in older adults, which could, in turn, offer greater neuroprotection for individuals at genetic risk for AD.

Another unique aspect of CYCLE-AD is the utilization of a consumer home-based exercise platform (Peloton Indoor Cycle), which offers many potential benefits to facilitate long-term exercise (Costello et al., 2011; Collado-Mateo et al., 2021). Sedentary older adults report feeling intimidated by fitness facilities and express concerns about keeping up with others in a group exercise setting (Costello et al., 2011); thus, a home-based intervention may serve as a facilitator for exercise in this cohort. Time is also frequently reported as a key barrier to initiating and maintaining consistent exercise (Costello et al., 2011); on-demand 24-h streaming exercise classes are predicted to enhance participant adherence by not requiring the participant to spend time traveling to a laboratory or exercise facility and follow a rigid class schedule. Supervision has been identified as another key aspect of exercise facilitation because it increases accountability, adherence, and quality of exercise performance (Collado-Mateo et al., 2021); the CYCLE AD exercise intervention, while delivered remotely, maintains a high level of oversight by using objective and frequent data monitoring. The exercise physiologist has remote access to the frequency, intensity, and duration of exercise and specific exercise performance variables (e.g., heart rate, cadence, power) from each exercise session on the Peloton portal. The use of objective exercise performance data removes the need for participant self-reported exercise adherence, which is well-documented to be highly variable and inaccurate (Prince et al., 2008; Hukkanen et al., 2018; Fiedler et al., 2021). Thus, this model of monitoring facilitates a shared decision-making approach between the exercise physiologist and participant, which is predicted to increase adherence to the study protocol and reduce potential study withdrawals.

Compared to continuous exercise training (CET; e.g., walking or running on a fixed incline treadmill at a fixed speed), virtually all Peloton classes, ranging from easy to difficult, involve high-intensity interval training (HIIT) in which the cycling instructors frequently ask the participants to vary the cadence, resistance, or both, enabling rapid fluctuations between low and moderate intensities or between moderate and high intensities. HIIT has three major advantages relative to CET: (1) HIIT is more efficient at improving fitness given the same exercise duration compared to CET (Gibala and McGee, 2008). (2) Frequent modulations of exercise intensity, as is accomplished with HIIT, reduce participant boredom, especially when instructions are delivered through an engaging and convenient streaming platform. (3) HIIT results in significantly greater improvements in memory functioning than CET in low-exercise healthy elders (Kovacevic et al., 2019). Thus, the capability to leverage the advantages of both HIIT and CET is anticipated to keep participants engaged and exercising at relatively high-intensity levels throughout the trial.

CYCLE-AD will test the efficacy of high-intensity aerobic exercise using an indoor cycle rather than a treadmill. Older adults prefer cycling to running (Reitlo et al., 2018) because the former does not involve whole-body load bearing, a key factor for elders with orthopedic injuries, arthritis, or chronic pain. In addition, unlike a treadmill, intensity transitions associated with HIIT can be accomplished quickly and efficiently on a cycle by varying cadence, resistance, or both. In contrast, treadmills are not quick and efficient in switching speed and incline angle.

Demonstrating positive results in the CYCLE-AD RCT will have a substantial impact on the field of AD. For older adults, the validation of an exercise program that could be initiated after decades of inactivity would empower them to take an active role in the prevention of AD. From a public health perspective, the identification of an exercise intervention that could contribute to delaying dementia onset by 5 years is estimated to reduce the prevalence of Alzheimer’s Disease and Related Diseases (ADRD) by 37%; delaying dementia by just 2 years reduces prevalence by 16% (Vickland et al., 2010). Our previously published data (Smith et al., 2011; Woodard et al., 2012; Smith et al., 2014; Smith et al., 2016) indicate that AD-related disease progression, as measured by memory testing and hippocampal volume, can be slowed in genetically high-risk individuals who exercise regularly. If successful, the CYCLE-AD trial has the potential to provide a scalable, low-cost intervention capable of substantially reducing healthcare costs by altering the disease trajectory of ADRD.

## Footnotes

1
https://www.onepeloton.com/bike

2
www.garmin.com

3
https://github.com/compimg/lst-ai
